# Paving the way for precision medicine v2.0 in intensive care by profiling necroinflammation in biofluids

**DOI:** 10.1038/s41418-018-0196-2

**Published:** 2018-09-10

**Authors:** Tom Vanden Berghe, Eric Hoste

**Affiliations:** 1 VIB Center for Inflammation Research, Ghent, Belgium; 2 Department of Biomedical Molecular Biology, Ghent University, Ghent, Belgium; 3 Division of Intensive Care, Department of Internal Medicine, Ghent University Hospital, Faculty of Medicine and Health Sciences, Ghent University, Ghent, Belgium; 4 Research Foundation Flanders, Brussels, Belgium

**Keywords:** Cell death and immune response, Predictive markers, Translational research

## Abstract

Current clinical diagnosis is typically based on a combination of approaches including clinical examination of the patient, clinical experience, physiologic and/or genetic parameters, high-tech diagnostic medical imaging, and an extended list of laboratory values mostly determined in biofluids such as blood and urine. One could consider this as precision medicine v1.0. However, recent advances in technology and better understanding of molecular mechanisms underlying disease will allow us to better characterize patients in the future. These improvements will enable us to distinguish patients who have similar clinical presentations but different cellular and molecular responses. Treatments will be able to be chosen more “precisely”, resulting in more appropriate therapy, precision medicine v2.0. In this review, we will reflect on the potential added value of recent advances in technology and a better molecular understanding of necrosis and inflammation for improving diagnosis and treatment of critically ill patients. We give a brief overview on the mutual interplay between necrosis and inflammation, which are two crucial detrimental factors in organ and/or systemic dysfunction. One of the challenges for the future will thus be the cellular and molecular profiling of necroinflammation in biofluids. The huge amount of data generated by profiling biomolecules and single cells through, for example, different omic-approaches is needed for data mining methods to allow patient-clustering and identify novel biomarkers. The real-time monitoring of biomarkers will allow continuous (re)evaluation of treatment strategies using machine learning models. Ultimately, we may be able to offer precision therapies specifically designed to target the molecular set-up of an individual patient, as has begun to be done in cancer therapeutics.

## Towards precision medicine v2.0 in critical care

Critical care mostly implies life-threatening situations involving systemic infection, inflammation and necrosis. Biofluids are an easily accessible source of liquid biopsies that can be used to monitor the evolution of the patient’s critical illness. The cellular and molecular profiling of necrosis and inflammation in biofluids using cutting-edge technologies such as realtime immunodiagnostics, next-generation sequencing and mass spectrometry will pave the way for precision medicine v2.0 in critical care. This is needed for data mining approaches to allow patientclustering, identify novel biomarkers and develop novel intervention strategies controlling necrosis and inflammation. The real-time monitoring of biomarkers will allow continued (re)evaluation of treatment strategies using machine learning models.

## Graphical abstract

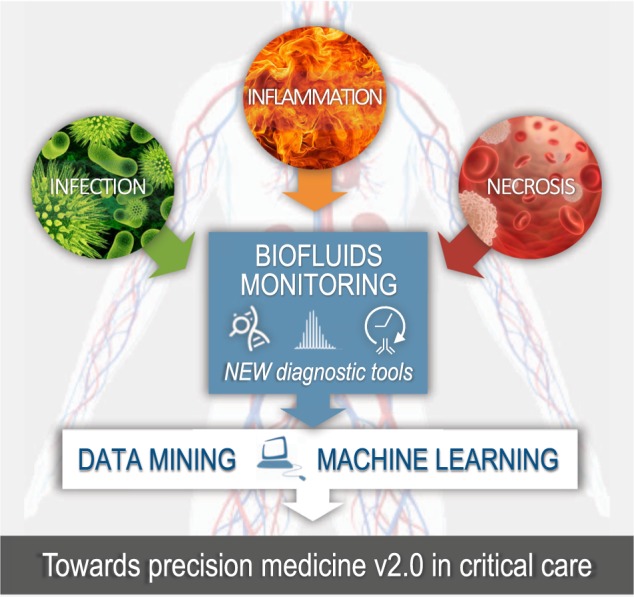

## Facts

- Necrosis and inflammation are two auto-amplifying detrimental factors in critically ill patients.
- Necrotic cells release damage-associated molecular patterns and chemo-/cytokines.
- Biomolecules released by necrotic cells and immune cells are circulating in biofluids of critically ill patients.
- The digitalization of monitoring intensive care patients allows data mining methods and machine learning models to finetune patient stratification and treatment strategies.

## Open questions

- Which circulating biomolecules and/or immune cell profiles have prognostic value for disease progression and mortality in critically ill patients?
- Is there therapeutic value in targeting novel biomarkers of necrosis or inflammation?
- How will we evolve to a patient-driven medical care, which allows a mutual secure interaction between biomedical (pre-)clinical research, health care services, and patients?
- How big will be the impact of data mining, artificial intelligence, and machine learning on reshaping critical care?

## Introduction

Patients with similar symptoms can have different diseases, and not all patients with the same disease respond equally to treatment [1]. To date, tailoring of medical treatment to the characteristics and needs of individual patients, or precision medicine, is predominately based on genetics. For example, the FDA recently approved four new cancer treatments and one treatment for cystic fibrosis for use in patients with specific genetic characteristics. The challenge of 21st century is to extend precision medicine beyond genetic stratification, by implementing novel molecular diagnostics and intervention strategies.

Critical illness is characterized by dysfunction of several organ systems, or multiple organ dysfunction syndrome (MODS), because of an inciting event—for instance, major trauma, surgery, or infection. This is explained by a dysregulated inflammatory stress response, which leads to a negative spiral where the effects of one organ dysfunction impacts on other organs. MODS often shows substantial individual variation in response to treatment due to individual genetic differences, co-morbidities, frailty, and dynamic disease fluctuations. More specifically, increased inflammation along immunosuppression and necrosis can occur dynamically and concurrently, originally coined as necroinflammation [2]. Therefore, dynamic monitoring of novel biomarkers for necrosis or inflammation is needed to stratify critically ill patients for treatment with new necrosis and/or inflammation intervention strategies [3]. The joined forces of different emerging fields such as real-time biomolecule diagnostics, single cell sequencing, the multiplicity of omics approaches, electronic health recording, data mining, and machine learning could potentially reshape profoundly the landscape of healthcare in the near future. Here, we will briefly review the current state of art on each of these topics related to necroinflammation.

## Necrosis (re)defined

Rudolf Virchow (1821‒1902, Prussia), founder of the Cell Theory (*Omnis cellula e cellula*) and cellular pathology, referred to tissue injury as “parenchymatous inflammation”. He postulated that tissue injury is caused by pathological changes within the cells. In 1858, he introduced the notion of cell death as the basis for pathology, with “necrobiosis” being a physiological process of spontaneous wearing out of living parts from the body and “necrosis” an accidental process. Virchow’s necrobiosis‒necrosis dichotomy resembles to some extent the current apoptosis‒necrosis classification [4]. Together with cellular and molecular insights into inflammation, came a shift into our understanding of the molecular interplay between cell death and inflammation at the site of tissue injury. This emerging field of research is crucial for understanding organismal homeostasis and how its processes contribute to a growing list of inflammatory and degenerative pathologies. Cell death is crucial as a mechanism for eliminating pathogens and regulating inflammation by exposing or releasing molecular patterns, but excessive cell death during inflammation is also one of the detrimental factors resulting in tissue damage [5].

For decades, apoptosis was considered as the standard cell death form during development, homeostasis, infection and pathogenesis, whereas necrosis was mostly considered as an “accidental” cell death in response to physico-chemical insults. An increasing amount of genetic evidence, as well as the discovery of chemical inhibitors of necrosis, have radically changed this view, and revealed the existence of multiple molecular pathways of necrosis [6]. The term “necrosis” comes from the Greek word “nekros”, which means “dead body”. Cellular necrosis is defined by rounding, swelling, cytoplasmic granulation, and plasma membrane rupture with consequent leakage of cellular contents into the extracellular space. Thus, the destruction of vital cellular functions is essentially the result of irreversible cell membrane damage. Multiple modes of necrosis (cell death) share these morphological hallmarks, and they are now examined for common or distinct underlying signaling pathways. Attempts to define and classify modes of necrosis and their underlying pathways have resulted in multiple neologisms, such as necroptosis, parthanatos, oxytosis/ferroptosis, (n)etosis, autoschizis, pyronecrosis, or pyroptosis emphasizing a particular aspect [6].

**Fig. 1:**
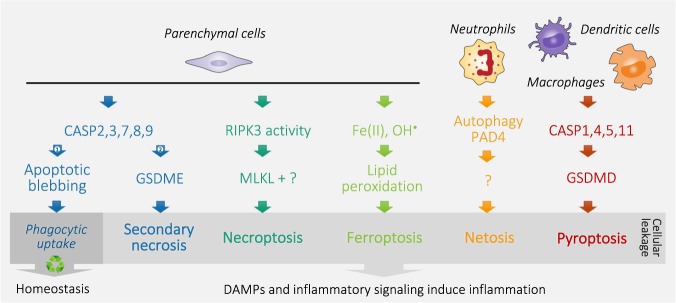
Schematic simplification of different modes of parenchymal and immune cell death. Organismal homeostasis is based on a balance between cell renewal and death, which is mediated by apoptosis. Apoptotic blebbing allows quick phagocytic uptake and recycling, which prevents leakage of the cellular content and subsequent inflammation (Arrow 1). In the absence or lack of sufficient phagocytic capacity (Arrow 2), apoptotic caspases cleave Gasdermin E (GSDME) resulting in cell rupture, referred to as secondary necrosis. Similarly, inflammatory caspases cleave Gasdermin D (GSDMD) to induce pyroptosis. Necroptosis is executed by the concerted action of RIPK3 kinase activity and the pseudokinase MLKL, whereas ferroptosis is fulfilled by free radical-induced lipid peroxidation catalyzed by Fe(II). Neutrophils typically die by netosis along expelling neutrophil extracellular traps (NETs), which is dependent on autophagy processes and PAD4-mediated citrullination. Different molecular mechanisms execute plasma membrane rupture, resulting in cellular leakage, defined as necrosis. Release of damage-associated molecular patterns (DAMPs) and inflammatory signaling by necrotic cells subsequently induce inflammation

In the human body, 1–5 million cells die every second. It is imperative that their clearance occurs efficiently and silently by phagocytes. This evolutionarily conserved process, termed efferocytosis, is critical to the maintenance of developmental and immune homeostasis [7]. As the goal of efferocytosis is the quiet removal of cellular corpses before the cells start to leak, one could theorize that part of the program of apoptosis would be the packaging of dying cells into immunologically inert pieces. However, in case of insufficient or absent phagocytic capacity, apoptotic cells, similar to necrotic cells, loose the integrity of the plasma membrane, referred to as secondary necrosis. Recently, the mechanism of action was found to be dependent on CASP3-dependent cleavage of Gasdermin E [8, 9]. This important finding might challenge the generally accepted dichotomy between non-leaky, immune-silent apoptosis and leaky, immunogenic necrosis. This view implies that apoptosis can be classified as a mode of necrosis (Fig. 1), with the notion that this stage of secondary necrosis is normally not reached in vivo owing to quick phagocytosis by neighboring cells or phagocytes.

## Necrosis-induced inflammatory response

For decades, the “self/non-self” model has been used as the sole framework to differentiate between homeostatic (that is, self and non-immunogenic) and pathogen-driven (that is, non-self and immunogenic) forms of cell death. However, the multitude of observations showing the propensity of endogenous entities to initiate an immune response illustrate the limitations of this model. Thus, the immune system has evolved to recognize, respond to, and remember danger in the form of damage-associated molecular patterns (DAMPs) or microbe-associated molecular patterns (MAMPs), previously referred to as pathogen-associated molecular patterns. This change in nomenclature was proposed because symbiotic flora and other non-pathogenic environmental microorganisms can also induce an immune-stimulatory response (for instance, upon disruption of intestinal epithelial barrier) [10], potentially boosting sepsis. For example, although lipopolysaccharide (LPS)-induced shock is generally considered a sterile shock model, antibiotics pretreatment can protect indicating the presence of a microbial component, probably caused by intestinal ischemia and barrier loss [11]. Cells dying under non-physiological conditions often reflects a pathological process, which is potentially dangerous to the host. The innate immune system developed mechanisms to detect this potential danger [12]. The ensuing acute inflammatory response rapidly delivers defenses that attempt to resolve the injurious process and repair the damage. Similarly, cell death will mobilize the adaptive immune system if immunogenic antigens are present.

For a long time, cell death has been misleadingly classified in a dichotomic manner. Thus, although apoptosis was considered to be a physiological, regulated, and non-immunogenic (or even tolerogenic), necrosis was viewed as a pathological, incontrollable and immunogenic variant of cellular demise [10]. Now it has become evident that such clear-cut differences do not exist. To date, research on immunogenic cell death is mainly performed in the context of pathogen defense and anticancer (immuno)therapy. From this field of research, we know that cell disruption induced by freeze–thaw is unable to activate dendritic cells in vitro [13] and fails to elicit protective immunity upon inoculation in syngeneic mice [14, 15]. This could imply that it is not merely cellular leakage that triggers an inflammatory response. The genetic programs of cell death can also actively transform DAMPs, altering their immunogenicity and dictating the effects of cell death on phagocytes and the immune response. This has fed the idea of the existence of at least one factor other than antigenicity that explains why some, but not all, forms of cell death are immunogenic. Therefore, similar to a vaccination procedure, it is proposed that immunogenicity depends on two key factors: antigenicity and adjuvanticity [10]. The presence of neo-antigens explains why dying cells can initiate an adaptive immune response provided that the cells also emit adjuvant signals as a consequence of cellular stress and death [16]. It is tempting to assume that (at least some) auto-immune disorders may originate from a situation in which an unwarranted wave of cell death in mistakenly perceived as immunogenic.

In addition, dying cells can also release chemo- and/or cytokines in a cell autonomous way through for example activation of nuclear factor -κB (NF-κB) that modulate the inflammatory response [17, 18]. An accumulating body of evidence shows also the implication of interleukin-1 (IL-1) family cytokines in initiating an inflammatory response to necrotic cells or cytotoxic stimuli [19]. Note that in the context of immunogenic anti-cancer therapies, the contribution of NF-κB-mediated inflammatory signaling is still a matter of debate [15, 20]. Conclusively, both processes, viz. DAMPs-induced immune responses and direct inflammatory signaling by necrotic cells, boost necroinflammation, and detrimentally contribute to disease progression. Therefore, identification of the key drivers of necrosis-initiated inflammation is likely to lead to major breakthroughs in the treatment of MODS.

## Inflammation-induced necrosis

Although the immune system has evolved to protect the host against infection, it is clear that responses can be generated under absolutely sterile conditions. This is painfully evident to anyone who has experienced blunt trauma (e.g., banging a thumb with a hammer) after which the affected site rapidly becomes inflamed. Trauma, bleeding, cell injury, and irritant particles are among the many kinds of sterile stimuli that can trigger various kinds of immune responses, including both innate and adaptive ones [21]. Thus, inflammation essentially occurs in response to infections as well as tissue injury, which results in permeabilizing local blood vessels to permit rapid ingress of neutrophils, monocytes, and blood-born molecules (such as complement, antibody, platelets, clotting factors, and acute phase reactants) in an attempt to resolve the dangerous situation. Cytokines and chemokines are key mediators of this inflammatory response, which causes quite some disturbance to tissue [19]. For example, infiltrating neutrophils contain a battery of destructive proteases that after degranulation are an important source of reactive oxygen species (ROS) and can expel web-like chromatin structures known as neutrophil extracellular traps (NETs) that neutralize and kill pathogens as a consequence of netosis [22]. In addition, high concentrations of ROS are injurious, because they oxidize protein, lipids, and damage the DNA. The resulting undesirable collateral tissue damage leads to further cell death and inflammation.

## An auto-amplifying loop between necrosis and inflammation drives MODS

Any disease that results in tissue injury increases the risk to develop MODS. Causal etiologies include infections, burns, severe trauma, and various other noninfectious inflammatory conditions. It is considered as one of the major causes of death in intensive care units (ICUs). The incidence of MODS in European ICU patients is increasing over the last decade from 39.7% in 2002 (SOAP study) to 51% in 2012 (ICON study) [23]. There are several proposed mechanisms to explain the pathophysiology of MODS [24]. A dysregulated immune response, or immune paralysis, in which the homeostasis between pro-inflammatory and anti-inflammatory reaction is lost is thought to be key in the development of MODS. This chronic failure propagates organ damage. The gut is thought to play an important role in MODS owing to surplus of inflammatory mediators, intestinal walls become hyperpermeable, which in turn, propagates the inflammatory response. Acute kidney injury (AKI) occurs in approximately half of ICU patients and is also a common complication in MODS associated with poor clinical outcomes [25, 26]. It is a syndrome that in the majority of ICU patients occurs as a consequence of disease (e.g., sepsis, trauma or shock), which evidently explains part of the observed morbidity and mortality. However, clinical data also show that AKI is not a mere innocent bystander, but also plays an important role in the prognosis of patients, as increasing severity of AKI also contributes to worse outcomes [26, 27]. To date, steroids are still one of the few treatment options for this dysregulated immune response in critically ill patients with MODS. It is tempting to speculate that the beneficial effects of steroid administration in critical care is likely due to its multitude of downstream targets in relation to necroinflammation [28]. However, large clinical studies on exogenous steroid administration are showing conflicting results [29], with some studies showing a mortality benefit [30, 31], whereas other could not demonstrate a beneficial effect [32, 33].

Recent data from basic and clinical research have begun to elucidate complex organ interactions in AKI between kidney and distant organs, including heart, lung, spleen, brain, liver, and gut [34]. The hypothesis of organ cross-talk and distant organ injury, often referred to as remote organ injury, has emerged over the last decade and may explain the reason for the potential negative impact of AKI on outcome [35]. Animal models clearly indicate that AKI induces distant organ dysfunction through different identified pathways, including inflammatory cascades, necrosis, induction of remote oxidative stress, and differential molecular expression [36]. Basically, the communication between different organs can only occur through transportation of biomolecules and immune cells in biofluids. This might also be a key detrimental factor in transplantation-induced distant organ injury [37]. This concept was, for example, illustrated in a rat allogeneic renal transplantation model, in which ischemic allografts (stored 24 h before transplantation), but not fresh immediately transplanted allografts, led to remote lung injury [38]. Pharmacological targeting of different modes of necrosis using a combined treatment with cyclosporine A, 3-aminobenzamide and necrostatin-1 attenuated lung injury. These experimental data suggest that DAMPs released from necrotic renal cells, mostly tubular cells, follow the circulation into the lung capillaries, where they harm the pulmonary tissue by two interconnected mechanisms: necrosis and inflammation [39], referred to as kidney–lung cross-talk in the critically ill patients [40]. Recently, it was found that this process is also enhanced by neutrophil extracellular traps and circulating histones [41]. A remote lung-injured transcriptome analysis also identified ischemia-specific changes that were distinguishable from those produced by uremia and involved several pro-inflammatory and proapoptotic pathways [42]. In summary, all these findings further strengthen the potential role of necroinflammation in remote organ damage.

A direct detrimental role for necrosis in MODS is also extensively shown using mouse experimental models reflecting systemic inflammatory response syndrome (SIRS), sepsis, and AKI. RIPK3-deficient, MLKL-deficient, and RIPK1 kinase death knockin mice are, to a different extent, protected against tumor necrosis factor (TNF)-induced SIRS [43–45]. A combined loss of CASP8 and RIPK3 provides a stronger protection against SIRS, but also kidney ischemia–reperfusion injury compared with loss of RIPK3 alone [43]. In kidney ischemia–reperfusion injury, different modes of necrosis act in a mutual way [43, 46], and ferroptosis of tubular kidney epithelium seems to be a dominant mode of cell death [47]. Although RIPK1 kinase inhibitors (Necrostatins) protects against TNF-induced SIRS [44, 48, 49], lipophilic radical traps such as ferrostatins or liproxstatins protect against AKI [47, 50]. Note that labile iron is a known risk factor to develop AKI in clinically relevant settings such as cardiac surgery-associated AKI, rhabdomyolysis-induced AKI and contrast-associated AKI [51, 52]. Iron chelation by desferoxamin has become a standard control agent for AKI when induced ex vivo in settings such as isolated renal tubules or in vivo in models of acute renal failure [53]. These data suggest that blocking cell death pathways could have therapeutic potential in context of SIRS and AKI.

There are also experimental data suggesting the therapeutic potential of targeting inflammation in sepsis. Mice deficient in the pathogen recognition receptors Toll-like receptor 4 or intracellular NOD-like receptor family member NLRP3 are protected against LPS-induced lethal shock [54–56]. Both receptors are required to induce the production of the inflammatory cytokines IL-1β and IL-18, which depends on the proteolytic activity of CASP1. Blocking pyroptosis by depleting mice from CASP11 also protects against LPS-induced shock [57]. A phenotypic in vivo screen revealed the superior therapeutic potential of neutralizing simultaneously IL-1 and IL-18 in sepsis rather than inhibiting the upstream inflammatory caspases CASP1 or -11 by using different experimental mouse models for septic shock [11]. In line with these data obtained in mice, patients with septic shock who did not survive displayed higher IL-18 levels than patients who survived [58, 59]. Also, in critically ill AKI patients, higher IL-18 levels were associated with non-recovery at day 60 and non-survival [60]. On the other hand, neutralization of IL-1 signaling using Kineret® (Anakinra; ILRa) in clinical trials resulted only in a marginal trend for increased survival [61], whereas IL-18 neutralization has not been evaluated in clinical studies so far [62]. These data obtained in septic mice and patients clearly underscore the need for patient stratification owing to the heterogeneity in the pathology of sepsis [63]. In addition to inflammasome-mediated pyroptosis and inflammatory signaling, evidence is increasing showing a potential detrimental role for NETs and/or netosis in MODS and AKI (reviewed by [22]).

**Fig. 2:**
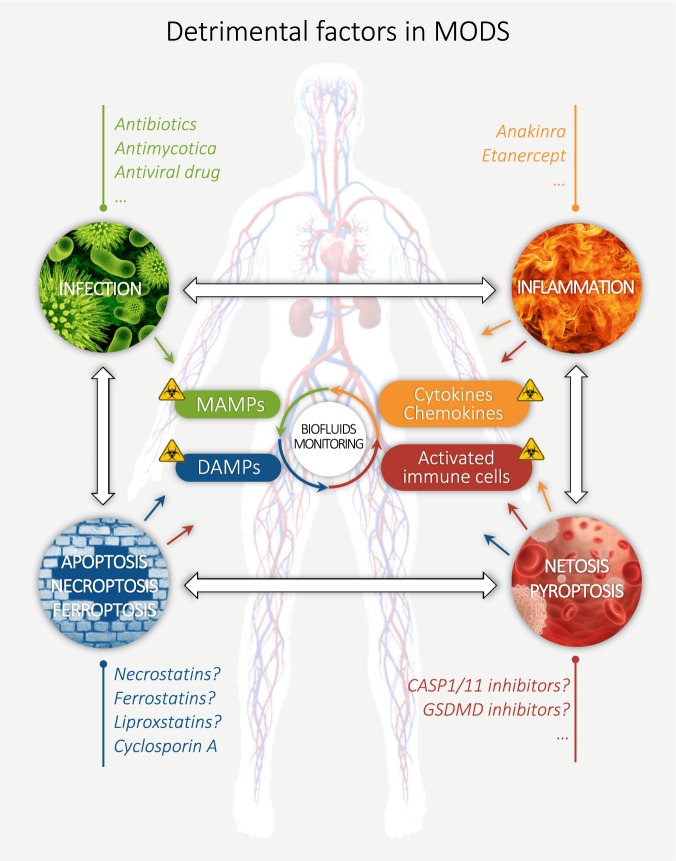
Hypothetical model representing major detrimental factors in multiple organ dysfunction syndrome (MODS). MODS mostly develops by the concerted action of four factors: (1) infection, (2) inflammation, (3) parenchymal necrosis (apoptosis, necroptosis, and ferroptosis), and (4) immune cell necrosis (pyroptosis and netosis). These features are responsible for the release of biomolecules (MAMPs, DAMPs, chemokines, and cytokines) in biofluids and the activation of immune cells, which both are often biohazardous worsening tissue damage. Monitoring this in biofluids will be crucial to stratify patients and identify novel potential biomarkers with predictive value. Ultimately, combined intervention strategies controlling infection, inflammation, and necrosis might be the key to effective treatment of MODS. MAMPs, microbe-associated molecular patterns; DAMPs, damage-associated molecular patterns

In summary, the experimental and preclinical support is increasing that indicates four not-exclusive key phenomena in the development of MODS: infection, inflammation, parenchymal cell necrosis, and immune cell necrosis (Fig. 2). These major processes result in the circulation of MAMPs, chemo- and cytokines, activated immune cells, and DAMPs, which in an auto-amplifying loop potentially cause distant organ injury. Monitoring these biomolecules and immune cells in biofluids will be crucial to stratify patients and identify novel potential biomarkers with predictive value. Ultimately, combined intervention strategies controlling infection, inflammation, and necrosis might be the key to effective treatment of MODS.

## Profiling necroinflammation in biofluids of critically ill ICU patients

**Fig. 3:**
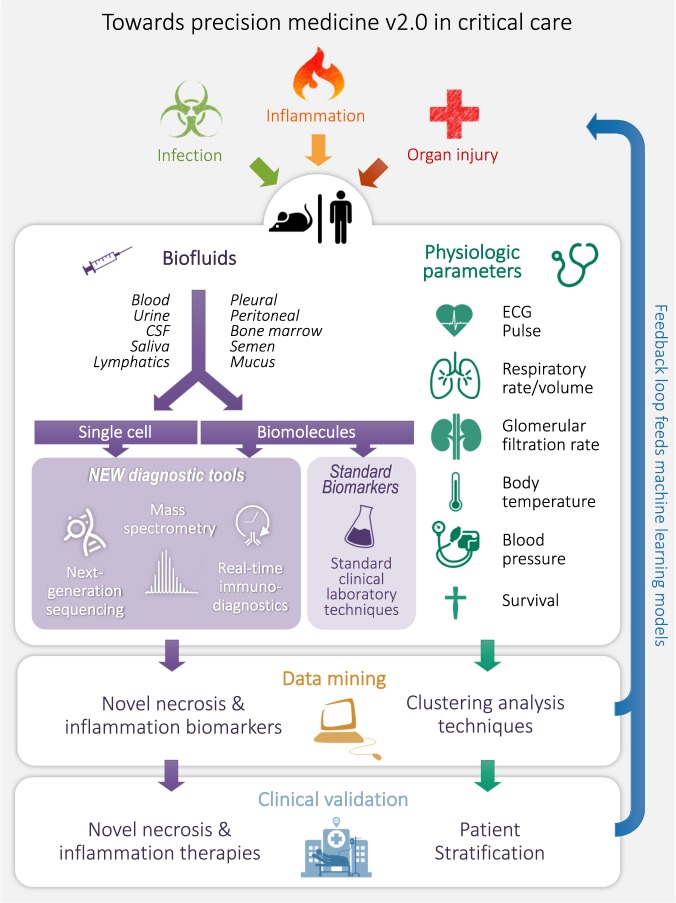
Schematic roadmap toward precision medicine v2.0 in critical care. Critical care mostly implies life-threatening situations involving systemic infection, inflammation, and organ dysfunction. Biofluids are an easily accessible source of liquid biopsies that can be used to monitor the evolution of the patient’s critical illness. To date, a clinical diagnosis is typically based on a combination of approaches including physiologic and/or genetic parameters, high-tech diagnostic medical imaging and an extended list of laboratory values determined in biofluids. Blood, urine, saliva, lymphatics, semen, mucus and cerebral spinal fluid (CSF), pleural, peritoneal, and bone marrow fluid are easily accessible biofluids. The cellular and molecular profiling of necrosis and inflammation in biofluids using cutting-edge technologies such as real-time immunodiagnostics, next-generation sequencing, and mass spectrometry will pave the way for precision medicine v2.0 in critical care. This is needed for data-mining approaches to allow patient-clustering, identify novel biomarkers, and develop novel intervention strategies controlling necrosis and inflammation. The real-time monitoring of biomarkers will allow continued (re)evaluation of treatment strategies using machine-learning models

In the ICU, the complexity and ambiguity of critical illness syndromes have been identified as fundamental justifications for the adoption of a precision approach to research and practice [64, 65]. This leads to considerable heterogeneity among patients, and conditions in which a “one size fits all” approach to therapy can lead to widely divergent results. Today a clinical diagnosis is typically based on a combination of elements including anamnesis, physiologic and/or genetic parameters, high-tech diagnostic medical imaging, and an extended list of laboratory values determined in biofluids such as blood and urine (Fig. 3). One could consider this as precision medicine v1.0. Experimental rodent models mimicking MODS unravel a still growing list of detrimental circulating biomolecules and immune cell profiles [63], which could be potentially novel biomarkers for stratification of critically ill patients. To pave the way for precision medicine v2.0, a joint venture between researchers and clinician will be crucial to daily monitor a panel of biomarkers in biofluids, to pinpoint correlations with survival and finally link an appropriate intervention strategy to the molecular diagnostic profiling. At present, AKI biomarkers have been successfully used to identify patients who may benefit from a so-called AKI bundle of care [66–68].

There are two problems at the basis of inconsistent translatability in critical care [69]. One problem is lack of reproducibility owing to false positive biomarker selection or no robust statistical models. The other, more importantly, is a lack of generalizability in moving from a narrow well-defined study population into broader applications in critical care. One way to improve trials is to focus on not just size but also heterogeneity. Dynamic disease fluctuations, for example, drive this heterogeneity, which might also partially explain the still disputed beneficial role of corticosteroids in critical care [70]. Therefore, daily monitoring of potentially novel biomarkers in biofluids is increasingly done to allow better patient stratification [71, 72]. Patients with similar clinical presentations typically have different cellular and molecular responses due to individual genetic differences and co-morbidities. To deal with this form of heterogeneity, an expanded list of novel biomarkers with predictive value is needed to allow determining subtypes of clinically similar patients. The list of potentially clinical relevant biomarkers is growing for sepsis [73] (Table 1) as well as AKI [74] (Table 2). Circulating DNA released form dying cells [75] or microorganisms [76], coined cell-free DNA (cf-DNA), is also gaining interest as a potential biomarker in MODS [3]. However, more work must be done in order to determine the origin of cf-DNA namely parenchymal cell necrosis versus immune cell necrosis [77, 78]. The translatability of these potentially novel biomarkers will critically depend on new technologies such as real-time immunodiagnostics to allow instant decision making [72].

**Table 1:** Potential biomarkers with predictive value for sepsis in critical illness

Target	Biofluid	Disease	Association	Ref.
HMGB1	Plasma	Severe blunt chest trauma	Risk of sepsis	[134]
	Plasma	Septic shock	Non-survival Higher APACHE II scores Increased pro-inflammatory cytokines	[135]
	Plasma	ARDS	ICU mortality	[136]
Cleaved cytokeratine 18	Plasma	Severe sepsis	Sepsis severity Mortality	[137]
	Plasma	Liver Transplantation	One-year non-survival	[138]
CASP3	Serum	Severe sepsis	Early mortality	[139]
sTREM	Blood	Septic shock	Non-survival	[140, 141]
DcR3	Serum	Sepsis	Biomarker sepsis	[142]
Procalcitonin	Serum	Critically ill	Adjunctive diagnostic marker to differentiate sepsis from SIRS	[143, 144]
IL-18	Plasma	ARDS	Morbidity and mortality	[145]
	Serum	Sepsis	Sepsis	[146, 147]
IL-18BP	Serum	Sepsis	Sepsis	[148]
Cytokine panel	Plasma	Sepsis	Mortality	[149]
Ang1	Plasma	Severe sepsis	Low levels predict poor outcome and high mortality risk	[150]
Ang2	Plasma	Severe sepsis	Organ dysfunction and injury	[150, 151]
Endocan	Plasma	Sepsis	Severity of illness and mortality	[152, 153]
Cell-free DNA	Plasma	Critically ill	Sepsis and mortality	[154, 155]
		Critically ill	No predictive value	[156]
Magnesium	Serum	Critically ill	Mortality	[157]
CHI3L1 (YKL40)	Serum	Sepsis	Sepsis	[158]

*Ang* Angiopoietins, *ARDS* acute respiratory distress syndrome, *CHI3L1* chitinase 3 like 1, *DcR3* soluble decoy receptor 3, *SLE* systemic lupus erythematosus, *sTREM-1* soluble triggering receptor expressed on myeloid cells 1

**Table 2:** Potential biomarkers with predictive value for acute kidney injury in critical illness

Target	Biofluid	Disease	Association	Ref.
AGT	Urine	ADHF	AKI	[159]
BPIFA2	Urine, blood	Critically ill	Early diagnosis of acute kidney injury	[160]
Calprotectin	Urine	Critically ill	Distinction between prerenal and intrinsic acute kidney injury	[161, 162]
CHI3L1 (YKL40)	Urine	Critically ill	Early diagnosis of acute kidney injury	[163, 164]
Cystatin C	Urine, plasma	Critically ill	Early diagnosis of acute kidney injury	[165, 166]
HSP72	Urine	Critically ill	Early diagnosis of acute kidney injury	[167]
IGFBP7	Urine	Critically ill	Early diagnosis of acute kidney injury	[168–170]
IL-18	Urine	Critically ill	Early diagnosis of acute kidney injury	[171]
	Urine	AKI	Mortality	[171]
	Urine	Cirrhosis	Diagnosis of acute tubular necrosis	[172]
	Urine	HIV	Proximal tubular dysfunction	[173]
KIM-1	Urine	Critically ill	Early diagnosis of acute kidney injury	[174]
L‑FABP	Urine, plasma	AKI	Mortality	[166, 175]
MCP-1	Urine	Cardiac surgery	AKI	[176]
microRNA	Urine	Cardiac surgery	Severe AKI and poor postoperative outcome	[177]
	Urine	Critically ill	AKI predisposition	[178]
NAG	Urine	Critically ill	Tubular damage	[179]
Netrin-1	Urine	Critically ill	Early diagnosis of acute kidney injury	[166, 180]
NGAL	Urine, plasma	Critically ill	Early diagnosis of acute kidney injury	[181, 182]
SBP-1	Urine	Critically ill	Early diagnosis of acute kidney injury	[183]
TIMP-2	Urine	Critically ill	Early diagnosis of acute kidney injury	[168–170]

*AGT* angiotensinogen, *ADHF* acute decompensated heart failure, *AKI* acute kidney injury, *BPIFA2* BPI fold-containing family A member 2, *CHI3L1* chitinase 3 like 1, *HIV* human immunodeficiency virus, *HSP* heat shock protein, *IGFBP7* insulin-like growth factor binding protein 7, *KIM-1* kidney injury molecule-1, *L-FABP* liver‑type fatty acid‑binding protein, *MCP-1* monocyte chemotactic protein 1, NAG *N*‑acetyl‑β‑d‑glucosaminidase; *NGAL* neutrophil gelatinase-associated lipocalin, *SBP-1* selenium-binding protein 1, *TIMP-2* tissue inhibitor of metalloproteinase 2

In addition to monitoring biomolecules as biomarkers, immune cell profiling has also resulted in potentially interesting biomarkers that need further validation. For example, prolonged lymphopenia [79, 80], CD64 expression on neutrophils [81, 82], Tregs increase [83, 84], prolonged depletion of dendritic cells [85], increased PD-1/PD-L1 expression on monocytes and neutrophils [86, 87], and increased BTLA expression on innate immune cell population [88] all have been shown to associate with disease severity and mortality. To date, unbiased -omics approaches to profile proteome [89], lipidome [90], glycome [91], metabolome [92], exome [93], and (epi-)genome [94, 95] are also extending precision oncology and start to be explored in ICU critical care in an attempt to create new predictive biomarkers [96] (Fig. 3). Profiling noncoding RNA might also have predictive value for disease severity and mortality. For example, long non-coding RNAs were investigated in sepsis [97, 98] and kidney injury [99], as well as miRNA profiles in sepsis [100, 101] and kidney injury [102]. Cutting-edge omics approaches such as oxidative proteomics [103], oxidative lipidomics [104], glycoprotomics [105], cellular glycomics [106], and single cell sequencing evolve quickly toward clinical diagnostic use. For example, single cell RNA sequencing [107], single cell genomics [108, 109], single cell epigenomics [110], single cell proteomics [111], single cell lipidomics [112], and single cell metabolomics [113] will undoubtedly be essential for profiling the differential immune cell responses in critically ill patients, and create prognostic value. Note that proteomics [114] and next-generation sequencing [76, 115] can also be used to identify the origin of MAMPs to diagnose the type of infection. It is however questionable if the current health economic evidence is high enough to support the more widespread use of whole-exome or -genome sequencing in clinical practice [116]. On the other hand, targeted sequencing or mass spectrometric analysis of robust biomarkers will probably become standard medical analysis techniques in clinical laboratory in the future. The justification for this expensive equipment will likely depend on the number of robust biomarkers, and their added value, e.g., the survival of the patient.

## How to deal with the data revolution in critical care?

“Big data in health” is defined by high volume, high diversity biological, clinical, environmental, and lifestyle information collected from single individuals to large cohorts, in relation to their health and wellness status, at one or several time points [117]. Big data come from a variety of sources, such as clinical trials, electronic health records, patient registries and databases, multidimensional data from genomic, epigenomic, transcriptomic, proteomic, metabolomic, and microbiomic measurements, and medical imaging. More recently, data are being integrated from social media, socioeconomic or behavioral indicators, occupational information, mobile applications, or environmental monitoring [118]. A major challenge for preclinical and clinical research is to obtain and achieve access to sufficient high quality, informative data. We need to progress from incomprehensible networks or ranking tables to a user-friendly and intuitive format.

Another major issue is the transferability of medical data between countries. Ownership of data by patients could overcome these obstacles. Presently, the patients do not have control over the access privileges to their medical records and remain unaware of the true value of the data they have. The USA have taken steps toward a “patient-driven economy” [119]. In such a scenario, the patient owns his/her data. By integrating the use of mobile devices, this could create a mutually interactive platform between biomedical (pre-)clinical research, health care services and patients through a world-standard public health record, although many challenges remain in achieving this [120]. For example, there is a need to have a much higher level of security than is possible today. One suggestion was to explore blockchain technology, which could be described as a distributed database that is used to maintain a continuously growing list of cryptographic records/blocks in a peer-to-peer network of users [121]. Originally used as the technology underlying “Bitcoin” to assure secure transactions, it might also be very suitable for application in healthcare. Essentially, projects fail more often because of the underappreciation of the complexities of ethical, legal, and social factors than for technological reasons. Data continue to increase at an exponential rate and the need for cross-border exchange of biomedical and healthcare data, cloud-storage, and cloud-computing is inevitable [122, 123]. Until many issues of data safety and security are solved, local solutions will be favored [124, 125].

## Data mining and machine learning models key to precision medicine 2.0?

A wealth of data are being collected in ICUs across the world, not only by standard clinical data management systems but also by clinical trials and researcher-driven clinical studies. These data need to be filtered from artifacts and standardized to a uniform readable format to allow clinical data mining approaches [126, 127]. Data mining is the process of pattern discovery and extraction where huge amount of data is involved. In the context of intensive care, this approach could be the key to identifying novel biomarkers and allow patient stratification (Fig. 3). One of the biggest benefits of the data-driven approach to biomarker discovery is the possibility of discovering novel pathobiology in the heterogeneity of critical illness compared to hypothesis-driven studies of familiar biomolecules. For example, data mining techniques are currently employed to try to predict mortality [128], one of the key issues in intensive care. Better patient stratification is also needed to improve the success rates of clinical trials, and critically depends on data mining methods including generalization, characterization, classification, clustering, association, evolution, pattern matching, data visualization, and meta-rule guided mining [129]. Dimensionality reduction and visualization techniques are exciting areas of research, which have the potential of redefining the single input monitoring approach currently applied in clinical practice. Looking even further forward, there is a need for integrative and interactive machine learning solutions, with teams of machine learning researchers and clinicians—who are directly involved in patient care and data acquisition—working in tandem to generate actionable insight and value from the increasingly large and complex critical care data [127]. Connecting daily monitoring of an increasing set of circulating biomolecules and immune cells in critically ill patients to data mining will feed machine-learning approaches. This form of artificial intelligence allows in a feedback loop continued reevaluation of novel patient stratification strategies and novel biomarkers/therapies targeting necrosis and inflammation (Fig. 3). In clinical practice, this approach will: (1) improve outcomes for individual patients through personalization of predictions, (2) allow earlier diagnosis and detection of adverse drug reactions, (3) provide better treatments and decision support for clinicians in cyclic processes, and (4) assist in understanding the progression of rare diseases. The multidimensional signatures will hopefully deliver a much higher predictive power than the single biomarkers used today. These improvements should eventually lead to lowered costs for the healthcare system.

## Conclusion and perspectives

Early advances in precision medicine have been illustrated in oncology, where both diagnosis and treatment are increasingly based on genomic features. Better success rates from the treatment of HER2-positive breast cancer [130] and EGFR-positive lung cancer [131] highlight the potential of precision medicine to lead to widespread changes in clinical practice. Growing interest is also reflected in new large-scale precision health projects, such as the NIH-sponsored Precision Medicine Initiative in the United States and the NHS-sponsored 100,000 Genomes project in Great Britain, as well as by citizen support for such ventures [132]. The promise of precision medicine is to have the right treatment for the right patient at the right time to maximize effectiveness [133]. In critical care, it will be important to follow a step-by-step procedure. For instance, try to answer urgent clinical questions first (such as best treatment option upon diagnosis of the type of infection), and then pose new ones that may not have been previously answerable (such as whether there are molecular subtypes in MODS, sepsis, or AKI). As omics and big data technologies proliferate, so too will studies utilizing them as biomarkers in critical illness (studying the genome, epigenome, transcriptome, proteome, metabolome, lipidome, microbiome,…). In all cases, we must remember the extreme heterogeneity of critical illness, and strive for generalizable disease-defining diagnostics and robust biomarkers that can help the entire spectrum of critical care research and delivery [69]. Ultimately, combined intervention strategies controlling infection, inflammation, and necrosis might be the key to effective treatment of MODS. It is not a matter if, but how quickly the landscape of intensive care will profoundly reshape. This will undoubtedly occur hand in hand along reshaping global health care. Although many challenges remain in achieving this, the evolution toward a patient-driven medical care, in which cloud-storage/computing and/or peer-to-peer technologies such as blockchain are needed, is probably inevitable. The role of mobile devices in this will definitely gain importance and could become a central player in providing a mutually interactive platform between biomedical (pre-)clinical research, health care services and patients.

## References

[1] Murugan R (2015). Movement towards personalised medicine in the ICU. Lancet Respir Med.

[2] Colombo M, Annoni G, Donato MF, Conte D, Martines D, Zaramella MG (1985). Serum type III procollagen peptide in alcoholic liver disease and idiopathic hemochromatosis: its relationship to hepatic fibrosis, activity of the disease and iron overload. Hepatology.

[3] Hofer S, Brenner T, Bopp C, Steppan J, Lichtenstern C, Weitz J (2009). Cell death serum biomarkers are early predictors for survival in severe septic patients with hepatic dysfunction. Crit Care.

[4] Virchow R. Cellular pathology: as based upon physiological and pathological histology: twenty lectures delivered in the Pathological Institute of Berlin During the Months of February, March and April, 1858. 1860.

[5] Wallach D, Kang TB, Kovalenko A (2014). Concepts of tissue injury and cell death in inflammation: a historical perspective. Nat Rev Immunol.

[6] Vanden Berghe T, Linkermann A, Jouan-Lanhouet S, Walczak H, Vandenabeele P (2014). Regulated necrosis: the expanding network of non-apoptotic cell death pathways. Nat Rev Mol Cell Biol.

[7] Kolb JP, Oguin TH, Oberst A, Martinez J (2017). Programmed cell death and inflammation: winter is coming. Trends Immunol.

[8] Rogers C, Fernandes-Alnemri T, Mayes L, Alnemri D, Cingolani G, Alnemri ES (2017). Cleavage of DFNA5 by caspase-3 during apoptosis mediates progression to secondary necrotic/pyroptotic cell death. Nat Commun.

[9] Wang Y, Gao W, Shi X, Ding J, Liu W, He H (2017). Chemotherapy drugs induce pyroptosis through caspase-3 cleavage of a gasdermin. Nature.

[10] Galluzzi L, Buque A, Kepp O, Zitvogel L, Kroemer G (2017). Immunogenic cell death in cancer and infectious disease. Nat Rev Immunol.

[11] Vanden Berghe T, Demon D, Bogaert P, Vandendriessche B, Goethals A, Depuydt B (2014). Simultaneous targeting of IL-1 and IL-18 is required for protection against inflammatory and septic shock. Am J Respir Crit Care Med.

[12] Matzinger P (2002). The danger model: a renewed sense of self. Science.

[13] Goldszmid RS, Idoyaga J, Bravo AI, Steinman R, Mordoh J, Wainstok R (2003). Dendritic cells charged with apoptotic tumor cells induce long-lived protective CD4+and CD8+T cell immunity against B16 melanoma. J Immunol (Baltim, Md: 1950).

[14] Casares N, Pequignot MO, Tesniere A, Ghiringhelli F, Roux S, Chaput N (2005). Caspase-dependent immunogenicity of doxorubicin-induced tumor cell death. J Exp Med.

[15] Aaes TL, Kaczmarek A, Delvaeye T, De Craene B, De Koker S, Heyndrickx L (2016). Vaccination with necroptotic cancer cells induces efficient anti-tumor immunity. Cell Rep.

[16] Shi Y, Zheng W, Rock KL (2000). Cell injury releases endogenous adjuvants that stimulate cytotoxic T cell responses. Proc Natl Acad Sci USA.

[17] Vanden Berghe T, Kalai M, Denecker G, Meeus A, Saelens X, Vandenabeele P (2006). Necrosis is associated with IL-6 production but apoptosis is not. Cell Signal.

[18] Kearney CJ, Martin SJ (2017). An inflammatory perspective on necroptosis. Mol Cell.

[19] Martin SJ (2016). Cell death and inflammation: the case for IL-1 family cytokines as the canonical DAMPs of the immune system. FEBS J.

[20] Yatim N, Jusforgues-Saklani H, Orozco S, Schulz O, Barreira da Silva R, Reis e Sousa C (2015). RIPK1 and NF-kappaB signaling in dying cells determines cross-priming of CD8(+) T cells. Science.

[21] Rock KL, Lai JJ, Kono H (2011). Innate and adaptive immune responses to cell death. Immunol Rev.

[22] Papayannopoulos V (2018). Neutrophil extracellular traps in immunity and disease. Nat Rev Immunol.

[23] Vincent JL, Lefrant JY, Kotfis K, Nanchal R, Martin-Loeches I, Wittebole X (2018). Comparison of European ICU patients in 2012 (ICON) versus 2002 (SOAP). Intensive Care Med.

[24] Pool R, Gomez H, Kellum JA (2018). Mechanisms of organ dysfunction in sepsis. Crit Care Clin.

[25] Hoste EAJ, Vandenberghe W (2017). Epidemiology of cardiac surgery-associated acute kidney injury. Best Pract Res Clin Anaesthesiol.

[26] Hoste EA, Bagshaw SM, Bellomo R, Cely CM, Colman R, Cruz DN (2015). Epidemiology of acute kidney injury in critically ill patients: the multinational AKI-EPI study. Intensive Care Med.

[27] Vaara ST, Pettila V, Kaukonen KM, Bendel S, Korhonen AM, Bellomo R (2014). The attributable mortality of acute kidney injury: a sequentially matched analysis*. Crit Care Med.

[28] Van Bogaert T, De Bosscher K, Libert C (2010). Crosstalk between TNF and glucocorticoid receptor signaling pathways. Cytokine Growth Factor Rev.

[29] Rochwerg B, Oczkowski SJ, Siemieniuk RAC, Agoritsas T, Belley-Cote E, D’Aragon F (2018). Corticosteroids in sepsis: an updated systematic review and meta-analysis. Crit Care Med.

[30] Annane D (2002). Effect of treatment with low doses of hydrocortisone and fludrocortisone on mortality in patients with septic shock. JAMA.

[31] Annane D, Renault A, Brun-Buisson C, Megarbane B, Quenot JP, Siami S (2018). Hydrocortisone plus fludrocortisone for adults with septic shock. N Engl J Med.

[32] Sprung CL, Annane D, Keh D, Moreno R, Singer M, Freivogel K (2008). Hydrocortisone therapy for patients with septic shock. N Engl J Med.

[33] Venkatesh B, Finfer S, Cohen J, Rajbhandari D, Arabi Y, Bellomo R (2018). Adjunctive glucocorticoid therapy in patients with septic shock. N Engl J Med.

[34] Doi K, Rabb H (2016). Impact of acute kidney injury on distant organ function: recent findings and potential therapeutic targets. Kidney Int.

[35] Depret F, Prud’homme M, Legrand M (2017). A role of remote organs effect in acute kidney injury outcome. Nephron.

[36] Grams ME, Rabb H (2012). The distant organ effects of acute kidney injury. Kidney Int.

[37] Vanden Berghe T, Linkermann A (2015). Take my breath away: necrosis in kidney transplants kills the lungs!. Kidney Int.

[38] Zhao H, Ning J, Lemaire A, Koumpa FS, Sun JJ, Fung A (2015). Necroptosis and parthanatos are involved in remote lung injury after receiving ischemic renal allografts in rats. Kidney Int.

[39] Linkermann A, Stockwell BR, Krautwald S, Anders HJ. Regulated cell death and inflammation: an auto-amplification loop causes organ failure. Nat Rev Immunol*.* 2014;14:759–67. 10.1038/nri3743 25324125

[40] Ko GJ, Rabb H, Hassoun HT (2009). Kidney-lung crosstalk in the critically ill patient. Blood Purif.

[41] Nakazawa D, Kumar SV, Marschner J, Desai J, Holderied A, Rath L (2017). Histones and neutrophil extracellular traps enhance tubular necrosis and remote organ injury in ischemic AKI. J Am Soc Nephrol.

[42] Hassoun HT, Grigoryev DN, Lie ML, Liu M, Cheadle C, Tuder RM (2007). Ischemic acute kidney injury induces a distant organ functional and genomic response distinguishable from bilateral nephrectomy. Am J Physiol Ren Physiol.

[43] Newton K, Dugger DL, Maltzman A, Greve JM, Hedehus M, Martin-McNulty B (2016). RIPK3 deficiency or catalytically inactive RIPK1 provides greater benefit than MLKL deficiency in mouse models of inflammation and tissue injury. Cell Death Differ.

[44] Duprez L, Takahashi N, Van Hauwermeiren F, Vandendriessche B, Goossens V, Vanden Berghe T (2011). RIP kinase-dependent necrosis drives lethal systemic inflammatory response syndrome. Immunity.

[45] Newton K, Dugger DL, Wickliffe KE, Kapoor N, de Almagro MC, Vucic D (2014). Activity of protein kinase RIPK3 determines whether cells die by necroptosis or apoptosis. Science.

[46] Linkermann A, Brasen JH, Darding M, Jin MK, Sanz AB, Heller JO (2013). Two independent pathways of regulated necrosis mediate ischemia-reperfusion injury. Proc Natl Acad Sci USA.

[47] Linkermann A, Skouta R, Himmerkus N, Mulay SR, Dewitz C, De Zen F (2014). Synchronized renal tubular cell death involves ferroptosis. Proc Natl Acad Sci USA.

[48] Degterev A, Maki JL, Yuan J (2013). Activity and specificity of necrostatin-1, small-molecule inhibitor of RIP1 kinase. Cell Death Differ.

[49] Takahashi N, Duprez L, Grootjans S, Cauwels A, Nerinckx W, DuHadaway JB (2012). Necrostatin-1 analogues: critical issues on the specificity, activity and in vivo use in experimental disease models. Cell Death Dis.

[50] Friedmann Angeli JP, Schneider M, Proneth B, Tyurina YY, Tyurin VA, Hammond VJ (2014). Inactivation of the ferroptosis regulator Gpx4 triggers acute renal failure in mice. Nat Cell Biol.

[51] Leaf DE, Rajapurkar M, Lele SS, Mukhopadhyay B, Rawn JD, Frendl G (2015). Increased plasma catalytic iron in patients may mediate acute kidney injury and death following cardiac surgery. Kidney Int.

[52] Leaf DE. Swinkels DW Catalytic iron and acute kidney injury. Am J Physiol Renal Physiol. 2016;311:F871–76. 10.1152/ajprenal.00388.2016 PMC5130458 27534995

[53] Sarhan M, von Massenhausen A, Hugo C, Oberbauer R, Linkermann A (2018). Immunological consequences of kidney cell death. Cell Death Dis.

[54] Hoshino K, Takeuchi O, Kawai T, Sanjo H, Ogawa T, Takeda Y (1999). Cutting edge: Toll-like receptor 4 (TLR4)-deficient mice are hyporesponsive to lipopolysaccharide: evidence for TLR4 as the Lps gene product. J Immunol.

[55] Qureshi ST, Lariviere L, Leveque G, Clermont S, Moore KJ, Gros P (1999). Endotoxin-tolerant mice have mutations in Toll-like receptor 4 (Tlr4). J Exp Med.

[56] Mariathasan S, Weiss DS, Newton K, McBride J, O’Rourke K, Roose-Girma M (2006). Cryopyrin activates the inflammasome in response to toxins and ATP. Nature.

[57] Kayagaki N, Warming S, Lamkanfi M, Vande Walle L, Louie S, Dong J (2011). Non-canonical inflammasome activation targets caspase-11. Nature.

[58] Oberholzer A, Harter L, Feilner A, Steckholzer U, Trentz O, Ertel W (2000). Differential effect of caspase inhibition on pro-inflammatory cytokine release in septic patients. Shock.

[59] Eidt MV, Nunes FB, Pedrazza L, Caeran G, Pellegrin G, Melo DA (2016). Biochemical and inflammatory aspects in patients with severe sepsis and septic shock: The predictive role of IL-18 in mortality. Clin Chim Acta.

[60] Murugan R, Wen X, Shah N, Lee M, Kong L, Pike F (2014). Plasma inflammatory and apoptosis markers are associated with dialysis dependence and death among critically ill patients receiving renal replacement therapy. Nephrol Dial Transplant.

[61] Opal SM, Fisher CJ, Jr., Dhainaut JF, Vincent JL, Brase R, Lowry SF, et al. Confirmatory interleukin-1 receptor antagonist trial in severe sepsis: a phase III, randomized, double-blind, placebo-controlled, multicenter trial. The Interleukin-1 Receptor Antagonist Sepsis Investigator Group. Crit Care Med. 1997;25: 1115–24. 10.1097/00003246-199707000-00010 9233735

[62] Dinarello CA, Fantuzzi G (2003). Interleukin-18 and host defense against infection. J Infect Dis.

[63] Sims CR, Nguyen TC, Mayeux PR (2016). Could biomarkers direct therapy for the septic patient?. J Pharmacol Exp Ther.

[64] Maslove DM, Lamontagne F, Marshall JC, Heyland DK (2017). A path to precision in the ICU. Crit Care.

[65] Seymour CW, Gomez H, Chang CH, Clermont G, Kellum JA, Kennedy J (2017). Precision medicine for all? Challenges and opportunities for a precision medicine approach to critical illness. Crit Care.

[66] Meersch M, Kullmar M, Schmidt C, Gerss J, Weinhage T, Margraf A (2018). Long-term clinical outcomes after early initiation of RRT in critically Ill patients with AKI. J Am Soc Nephrol.

[67] Meersch M, Schmidt C, Hoffmeier A, Van Aken H, Wempe C, Gerss J (2017). Prevention of cardiac surgery-associated AKI by implementing the KDIGO guidelines in high risk patients identified by biomarkers: the PrevAKI randomized controlled trial. Intensive Care Med.

[68] Gocze I, Jauch D, Gotz M, Kennedy P, Jung B, Zeman F, et al. Biomarker-guided intervention to prevent acute kidney injury after major surgery: the prospective randomized BigpAK study. Ann Surg. 2017;267:1013–20. 10.1097/SLA.0000000000002485 28857811

[69] Sweeney TE, Khatri P (2017). Generalizable biomarkers in critical care: toward precision medicine. Crit Care Med.

[70] Rochwerg B, Oczkowski S, Siemieniuk RA, Menon K, Szczeklik W, English S (2017). Corticosteroids in sepsis: an updated systematic review and meta-analysis (protocol). BMJ Open.

[71] Iapichino G, Marzorati S, Umbrello M, Baccalini R, Barassi A, Cainarca M (2010). Daily monitoring of biomarkers of sepsis in complicated long-term ICU-patients: can it support treatment decisions?. Minerva Anestesiol.

[72] Zirath H, Schnetz G, Glatz A, Spittler A, Redl H, Peham JR. Bedside immune monitoring: an automated immunoassay platform for rapid quantification of blood biomarkers in patient serum. Anal Chem. 2017;89:4817–23. 10.1021/acs.analchem.6b03624 28382820

[73] Biron BM, Ayala A, Lomas-Neira JL (2015). Biomarkers for Sepsis: what Is and What Might Be?. Biomark Insights.

[74] Prowle JR (2015). Measurement of AKI biomarkers in the ICU: still striving for appropriate clinical indications. Intensive Care Med.

[75] Harrington JS, Choi AMK, Nakahira K (2017). Mitochondrial DNA in Sepsis. Curr Opin Crit Care.

[76] Grumaz S, Stevens P, Grumaz C, Decker SO, Weigand MA, Hofer S (2016). Next-generation sequencing diagnostics of bacteremia in septic patients. Genome Med.

[77] Margraf S, Logters T, Reipen J, Altrichter J, Scholz M, Windolf J (2008). Neutrophil-derived circulating free DNA (cf-DNA/NETs): a potential prognostic marker for posttraumatic development of inflammatory second hit and sepsis. Shock.

[78] Altrichter J, Zedler S, Kraft R, Faist E, Mitzner SR, Sauer M (2010). Neutrophil-derived circulating free DNA (cf-DNA/NETs), a potential prognostic marker for mortality in patients with severe burn injury. Eur J Trauma Emerg Surg.

[79] de Jager CPC, van Wijk PTL B, Mathoera Rejiv, de Jongh-Leuvenink Jacqueline, van der Poll Tom, C Wever Peter. Lymphocytopenia and neutrophil-lymphocyte count ratio predict bacteremia better than conventional infection markers in an emergency care unit. *Crit Care.* 2010;14:R!92. 10.1186/cc9309 PMC3219299 21034463

[80] Drewry AM, Samra N, Skrupky LP, Fuller BM, Compton SM, Hotchkiss RS (2014). Persistent lymphopenia after diagnosis of sepsis predicts mortality. Shock.

[81] Chen QQ, Shi JF, Fei AH, Wang FL, Pan SM, Wang WW (2014). Neutrophil CD64 expression is a predictor of mortality for patients in the intensive care unit. Int J Clin Exp Pathol.

[82] Farias MG, de Lucena NP, Dal Bo S, de Castro SM (2014). Neutrophil CD64 expression as an important diagnostic marker of infection and sepsis in hospital patients. J Immunol Methods.

[83] Chen K, Zhou QX, Shan HW, Li WF, Lin ZF (2015). Prognostic value of CD4(+)CD25(+) Tregs as a valuable biomarker for patients with sepsis in ICU. World J Emerg Med.

[84] Huang H, Xu R, Lin F, Bao C, Wang S, Ji C (2015). High circulating CD39(+) regulatory T cells predict poor survival for sepsis patients. Int J Infect Dis.

[85] Grimaldi D, Louis S, Pene F, Sirgo G, Rousseau C, Claessens YE (2011). Profound and persistent decrease of circulating dendritic cells is associated with ICU-acquired infection in patients with septic shock. Intensive Care Med.

[86] Huang X, Chen Y, Chung CS, Yuan Z, Monaghan SF, Wang F (2014). Identification of B7-H1 as a novel mediator of the innate immune/pro-inflammatory response as well as a possible myeloid cell prognostic biomarker in sepsis. J Immunol.

[87] Guignant C, Lepape A, Huang X, Kherouf H, Denis L, Poitevin F (2011). Programmed death-1 levels correlate with increased mortality, nosocomial infection and immune dysfunctions in septic shock patients. Crit Care.

[88] Shubin NJ, Chung CS, Heffernan DS, Irwin LR, Monaghan SF, Ayala A (2012). BTLA expression contributes to septic morbidity and mortality by inducing innate inflammatory cell dysfunction. J Leukoc Biol.

[89] Geyer PE, Kulak NA, Pichler G, Holdt LM, Teupser D, Mann M (2016). Plasma proteome profiling to assess human health and disease. Cell Syst.

[90] Ghosh A, Nishtala K (2017). Biofluid lipidome: a source for potential diagnostic biomarkers. Clin Transl Med.

[91] Gudelj I, Baciarello M, Ugrina I, De Gregori M, Napolioni V, Ingelmo PM (2016). Changes in total plasma and serum N-glycome composition and patient-controlled analgesia after major abdominal surgery. Sci Rep.

[92] Ferrario M, Cambiaghi A, Brunelli L, Giordano S, Caironi P, Guatteri L (2016). Mortality prediction in patients with severe septic shock: a pilot study using a target metabolomics approach. Sci Rep.

[93] Meng L, Pammi M, Saronwala A, Magoulas P, Ghazi AR, Vetrini F (2017). Use of exome sequencing for infants in intensive care units: ascertainment of severe single-gene disorders and effect on medical management. JAMA Pediatr.

[94] Lu Y, Li S, Zhu S, Gong Y, Shi J, Xu L (2017). Methylated DNA/RNA in body fluids as biomarkers for lung cancer. Biol Proced Online.

[95] Sweeney TE, Shidham A, Wong HR, Khatri P (2015). A comprehensive time-course-based multicohort analysis of sepsis and sterile inflammation reveals a robust diagnostic gene set. Sci Transl Med.

[96] Russell JA, Spronk P, Walley KR Using multiple ‘omics strategies for novel therapies in sepsis. Intensive Care Med. 2018;44:509–11. 10.1007/s00134-018-5122-z 29546534

[97] Ho J, Chan H, Wong SH, Wang MH, Yu J, Xiao Z (2016). The involvement of regulatory non-coding RNAs in sepsis: a systematic review. Crit Care.

[98] Zhang TN, Li D, Xia J, Wu QJ, Wen R, Yang N (2017). Non-coding RNA: a potential biomarker and therapeutic target for sepsis. Oncotarget.

[99] Lin J, Zhang X, Xue C, Zhang H, Shashaty MG, Gosai SJ (2015). The long noncoding RNA landscape in hypoxic and inflammatory renal epithelial injury. Am J Physiol Ren Physiol.

[100] Vasilescu C, Dragomir M, Tanase M, Giza D, Purnichescu-Purtan R, Chen M (2017). Circulating miRNAs in sepsis-A network under attack: an in-silico prediction of the potential existence of miRNA sponges in sepsis. PLoS ONE.

[101] Caserta S, Kern F, Cohen J, Drage S, Newbury SF, Llewelyn MJ (2016). Circulating Pplasma microRNAs can differentiate human sepsis and systemic inflammatory response syndrome (SIRS). Sci Rep.

[102] Xie JX, Fan X, Drummond CA, Majumder R, Xie Y, Chen T (2017). MicroRNA profiling in kidney disease: plasma versus plasma-derived exosomes. Gene.

[103] Lourenco dos Santos S, Baraibar MA, Lundberg S, Eeg-Olofsson O, Larsson L, Friguet B (2015). Oxidative proteome alterations during skeletal muscle ageing. Redox Biol.

[104] Spickett CM, Pitt AR (2015). Oxidative lipidomics coming of age: advances in analysis of oxidized phospholipids in physiology and pathology. Antioxid Redox Signal.

[105] Yang S, Chatterjee S, Cipollo J. The glycoproteomics-mass spectrometry for studying glycosylation in cardiac hypertrophy and heart failure. Proteomics Clin Appl. 2018: 1700075. 10.1002/prca.201700075 29424483

[106] Fujitani N, Furukawa J, Araki K, Fujioka T, Takegawa Y, Piao J (2013). Total cellular glycomics allows characterizing cells and streamlining the discovery process for cellular biomarkers. Proc Natl Acad Sci USA.

[107] Picelli S (2017). Single-cell RNA-sequencing: the future of genome biology is now. RNA Biol.

[108] Gawad C, Koh W, Quake SR (2016). Single-cell genome sequencing: current state of the science. Nat Rev Genet.

[109] Giladi A, Amit I (2018). Single-cell genomics: a stepping stone for future immunology discoveries. Cell.

[110] Kelsey G, Stegle O, Reik W (2017). Single-cell epigenomics: recording the past and predicting the future. Science.

[111] Budnik B, Levy E, Harmange G, Slavov N. Mass-spectrometry of single mammalian cells quantifies proteome heterogeneity during cell differentiation. *bioRxiv* 2018: 102681. 10.1186/s13059-018-1547-5 PMC6196420 30343672

[112] Passarelli MK, Ewing AG, Winograd N (2013). Single-cell lipidomics: characterizing and imaging lipids on the surface of individual Aplysia californica neurons with cluster secondary ion mass spectrometry. Anal Chem.

[113] Zenobi R (2013). Single-cell metabolomics: analytical and biological perspectives. Science.

[114] Malmstrom E, Kilsgard O, Hauri S, Smeds E, Herwald H, Malmstrom L (2016). Large-scale inference of protein tissue origin in gram-positive sepsis plasma using quantitative targeted proteomics. Nat Commun.

[115] Rato S, Golumbeanu M, Telenti A, Ciuffi A (2017). Exploring viral infection using single-cell sequencing. Virus Res.

[116] Schwarze K, Buchanan J, Taylor JC, Wordsworth S. Are whole-exome and whole-genome sequencing approaches cost-effective? A systematic review of the literature. Genet Med. 2018. 10.1038/gim.2017.247 29446766

[117] Auffray C, Balling R, Barroso I, Bencze L, Benson M, Bergeron J (2016). Making sense of big data in health research: towards an EU action plan. Genome Med.

[118] Fernandez-Luque L, Bau T (2015). Health and social media: perfect storm of information. Healthc Inform Res.

[119] Mandl KD, Kohane IS (2016). Time for a patient-driven health information economy?. N Engl J Med.

[120] Roehrs A, da Costa CA, Righi RD, de Oliveira KS (2017). Personal health records: a systematic literature review. J Med Internet Res.

[121] Mamoshina P, Ojomoko L, Yanovich Y, Ostrovski A, Botezatu A, Prikhodko P (2018). Converging blockchain and next-generation artificial intelligence technologies to decentralize and accelerate biomedical research and healthcare. Oncotarget.

[122] Hucikova A, Babic A (2016). Overcoming constraints in healthcare with cloud technology. Stud Health Technol Inform.

[123] Kruse CS, Frederick B, Jacobson T, Monticone DK (2017). Cybersecurity in healthcare: a systematic review of modern threats and trends. Technol Health Care.

[124] Griebel L, Prokosch HU, Kopcke F, Toddenroth D, Christoph J, Leb I (2015). A scoping review of cloud computing in healthcare. BMC Med Inform Decis Mak.

[125] De Moor G, Sundgren M, Kalra D, Schmidt A, Dugas M, Claerhout B (2015). Using electronic health records for clinical research: the case of the EHR4CR project. J Biomed Inform.

[126] Zhang Y, Guo SL, Han LN, Li TL (2016). Application and exploration of big data mining in clinical medicine. Chin Med J (Engl).

[127] Johnson AE, Ghassemi MM, Nemati S, Niehaus KE, Clifton DA, Clifford GD (2016). Machine learning and decision support in critical care. Proc IEEE Inst Electr Electron Eng.

[128] Navaz A, Mohammed E, Serhani M, Zaki N. The use of data mining techniques to predict mortality and length of stay in an ICU. 2016 12th International Conference on Innovations in Information Technology (IIT) 2016:1–5.

[129] Valdes G, Luna JM, Eaton E, Simone CB, Ungar LH, Solberg TD (2016). MediBoost: a patient stratification tool for interpretable decision making in the era of precision medicine. Sci Rep.

[130] Moasser MM, Krop IE (2015). The evolving landscape of HER2 targeting in breast cancer. JAMA Oncol.

[131] Maemondo M, Inoue A, Kobayashi K, Sugawara S, Oizumi S, Isobe H (2010). Gefitinib or chemotherapy for non-small-cell lung cancer with mutated EGFR. N Engl J Med.

[132] Kaufman DJ, Baker R, Milner LC, Devaney S, Hudson KL (2016). A survey of U.S adults’ opinions about conduct of a nationwide precision medicine initiative(R) cohort study of genes and environment. PLoS ONE.

[133] Hudson K, Lifton R, Initiative P-L-B. *The precision medicine initiative cohort program—Building a Research Foundation for 21st Century Medicine.* Precision Medicine Initiative (PMI) Working Group Report to the Advisory Committee to the Director, NIH. 2015.

[134] Wang XW, Karki A, Zhao XJ, Xiang XY, Lu ZQ (2014). High plasma levels of high mobility group box 1 is associated with the risk of sepsis in severe blunt chest trauma patients: a prospective cohort study. J Cardiothorac Surg.

[135] Stevens NE, Chapman MJ, Fraser CK, Kuchel TR, Hayball JD, Diener KR (2017). Therapeutic targeting of HMGB1 during experimental sepsis modulates the inflammatory cytokine profile to one associated with improved clinical outcomes. Sci Rep.

[136] Tseng CC, Fang WF, Leung SY, Chen HC, Chang YC, Wang CC (2014). Impact of serum biomarkers and clinical factors on intensive care unit mortality and 6-month outcome in relatively healthy patients with severe pneumonia and acute respiratory distress syndrome. Dis Markers.

[137] Lorente L, Martin MM, Perez-Cejas A, Lopez RO, Ferreres J, Sole-Violan J (2017). Higher serum caspase-cleaved cytokeratin-18 levels during the first week of sepsis diagnosis in non-survivor patients. Clin Chem Lab Med.

[138] Lorente L, Rodriguez ST, Sanz P, Perez-Cejas A, Padilla J, Diaz D (2016). Prognostic value of serum caspase-cleaved cytokeratin-18 levels before liver transplantation for one-year survival of patients with hepatocellular carcinoma. Int J Mol Sci.

[139] Lorente L, Martin MM, Ferreres J, Sole-Violan J, Labarta L, Diaz C (2016). Serum caspase 3 levels are associated with early mortality in severe septic patients. J Crit Care.

[140] Brenner T, Uhle F, Fleming T, Wieland M, Schmoch T, Schmitt F (2017). Soluble TREM-1 as a diagnostic and prognostic biomarker in patients with septic shock: an observational clinical study. Biomarkers.

[141] Gibot S, Kolopp-Sarda MN, Bene MC, Cravoisy A, Levy B, Faure GC (2004). Plasma level of a triggering receptor expressed on myeloid cells-1: its diagnostic accuracy in patients with suspected sepsis. Ann Intern Med.

[142] Kim S, Mi L, Zhang L (2012). Specific elevation of DcR3 in sera of sepsis patients and its potential role as a clinically important biomarker of sepsis. Diagn Microbiol Infect Dis.

[143] O’Grady NP, Barie PS, Bartlett JG, Bleck T, Carroll K, Kalil AC. Guidelines for evaluation of new fever in critically ill adult patients: 2008 update from the American College of Critical Care Medicine and the Infectious Diseases Society of America. Crit Care Med. 2008;36:1330–49. 10.1097/CCM.0b013e318169eda9 18379262

[144] Sridharan P, Chamberlain RS (2013). The efficacy of procalcitonin as a biomarker in the management of sepsis: slaying dragons or tilting at windmills?. Surg Infect (Larchmt).

[145] Dolinay T, Kim YS, Howrylak J, Hunninghake GM, An CH, Fredenburgh L (2012). Inflammasome-regulated cytokines are critical mediators of acute lung injury. Am J Respir Crit Care Med.

[146] Endo S, Inada K, Yamada Y, Wakabayashi G, Ishikura H, Tanaka T (2000). Interleukin 18 (IL-18) levels in patients with sepsis. J Med.

[147] Grobmyer SR, Lin E, Lowry SF, Rivadeneira DE, Potter S, Barie PS (2000). Elevation of IL-18 in human sepsis. J Clin Immunol.

[148] Novick D, Schwartsburd B, Pinkus R, Suissa D, Belzer I, Sthoeger Z (2001). A novel IL-18BP ELISA shows elevated serum IL-18BP in sepsis and extensive decrease of free IL-18. Cytokine.

[149] Mera S, Tatulescu D, Cismaru C, Bondor C, Slavcovici A, Zanc V (2011). Multiplex cytokine profiling in patients with sepsis. APMIS.

[150] Ricciuto DR, dos Santos CC, Hawkes M, Toltl LJ, Conroy AL, Rajwans N (2011). Angiopoietin-1 and angiopoietin-2 as clinically informative prognostic biomarkers of morbidity and mortality in severe sepsis. Crit Care Med.

[151] Orfanos SE, Kotanidou A, Glynos C, Athanasiou C, Tsigkos S, Dimopoulou I (2007). Angiopoietin-2 is increased in severe sepsis: correlation with inflammatory mediators. Crit Care Med.

[152] Scherpereel A, Depontieu F, Grigoriu B, Cavestri B, Tsicopoulos A, Gentina T (2006). Endocan, a new endothelial marker in human sepsis. Crit Care Med.

[153] Palud A, Parmentier-Decrucq E, Pastre J, De Freitas Caires N, Lassalle P, Mathieu D (2015). Evaluation of endothelial biomarkers as predictors of organ failures in septic shock patients. Cytokine.

[154] Rhodes A, Wort SJ, Thomas H, Collinson P, Bennett ED, Plasma DNA (2006). concentration as a predictor of mortality and sepsis in critically ill patients. Crit Care.

[155] Saukkonen K, Lakkisto P, Varpula M, Varpula T, Voipio-Pulkki LM, Pettila V (2007). Association of cell-free plasma DNA with hospital mortality and organ dysfunction in intensive care unit patients. Intensive Care Med.

[156] Garnacho-Montero J, Huici-Moreno MJ, Gutierrez-Pizarraya A, Lopez I, Marquez-Vacaro JA, Macher H (2014). Prognostic and diagnostic value of eosinopenia, C-reactive protein, procalcitonin, and circulating cell-free DNA in critically ill patients admitted with suspicion of sepsis. Crit Care.

[157] Reis AMD, Fruchtenicht AVG, Athaydes LC, Loss S, Moreira LF (2017). Biomarkers as predictors of mortality in critically ill patients with solid tumors. Acad Bras Cienc.

[158] Hattori N, Oda S, Sadahiro T, Nakamura M, Abe R, Shinozaki K (2009). YKL-40 identified by proteomic analysis as a biomarker of sepsis. Shock.

[159] Yang X, Chen C, Tian J, Zha Y, Xiong Y, Sun Z (2015). Urinary angiotensinogen level predicts AKI in acute decompensated heart failure: a prospective, two-stage study. J Am Soc Nephrol.

[160] Kota SK, Pernicone E, Leaf DE, Stillman IE, Waikar SS, Kota SB (2017). BPI fold-containing family a member 2/parotid secretory protein is an early biomarker of AKI. J Am Soc Nephrol.

[161] Heller F, Frischmann S, Grunbaum M, Zidek W, Westhoff TH (2011). Urinary calprotectin and the distinction between prerenal and intrinsic acute kidney injury. Clin J Am Soc Nephrol.

[162] Seibert FS, Pagonas N, Arndt R, Heller F, Dragun D, Persson P (2013). Calprotectin and neutrophil gelatinase–associated lipocalin in the differentiation of pre‐renal and intrinsic acute kidney injury. Acta Physiol.

[163] De Loor J, Decruyenaere J, Demeyere K, Nuytinck L, Hoste EA, Meyer E (2016). Urinary chitinase 3-like protein 1 for early diagnosis of acute kidney injury: a prospective cohort study in adult critically ill patients. Crit Care.

[164] De Loor J, Herck I, Francois K, Van Wesemael A, Nuytinck L, Meyer E (2017). Diagnosis of cardiac surgery-associated acute kidney injury: differential roles of creatinine, chitinase 3-like protein 1 and neutrophil gelatinase-associated lipocalin: a prospective cohort study. Ann Intensive Care.

[165] Nakhjavan-Shahraki B, Yousefifard M, Ataei N, Baikpour M, Ataei F, Bazargani B (2017). Accuracy of cystatin C in prediction of acute kidney injury in children; serum or urine levels: which one works better? A systematic review and meta-analysis. BMC Nephrol.

[166] Beker BM, Corleto MG, Fieiras C, Musso CG (2018). Novel acute kidney injury biomarkers: their characteristics, utility and concerns. Int Urol Nephrol.

[167] Morales-Buenrostro LE, Salas-Nolasco OI, Barrera-Chimal J, Casas-Aparicio G, Irizar-Santana S, Perez-Villalva R (2014). Hsp72 is a novel biomarker to predict acute kidney injury in critically ill patients. PLoS ONE.

[168] Meersch M, Schmidt C, Van Aken H, Martens S, Rossaint J, Singbartl K (2014). Urinary TIMP-2 and IGFBP7 as early biomarkers of acute kidney injury and renal recovery following cardiac surgery. PLoS ONE.

[169] Wetz AJ, Richardt EM, Wand S, Kunze N, Schotola H, Quintel M (2015). Quantification of urinary TIMP-2 and IGFBP-7: an adequate diagnostic test to predict acute kidney injury after cardiac surgery?. Crit Care.

[170] Pilarczyk K, Edayadiyil-Dudasova M, Wendt D, Demircioglu E, Benedik J, Dohle DS (2015). Urinary [TIMP-2]*[IGFBP7] for early prediction of acute kidney injury after coronary artery bypass surgery. Ann Intensive Care.

[171] Nisula S, Yang R, Poukkanen M, Vaara ST, Kaukonen KM, Tallgren M (2015). Predictive value of urine interleukin-18 in the evolution and outcome of acute kidney injury in critically ill adult patients. Br J Anaesth.

[172] Puthumana J, Ariza X, Belcher JM, Graupera I, Gines P, Parikh CR (2017). Urineinterleukin 18 and lipocalin 2 are biomarkers of acute tubular necrosis in patients with cirrhosis: a systematic review and meta-analysis. Clin Gastroenterol Hepatol.

[173] DOrluwene CG, Deebii N, Odum EP. Urinary Interleukin (Il)-18 as an early predictive biomarker of subclinical proximal tubular dysfunction in HIV-infected patients Exposed to Tenofovir. J AIDS Clin Res. 2015;6:497.

[174] Yin C, Wang N. Kidney injury molecule-1 in kidney disease. Ren Fail. 2016;38:1567–73. 10.1080/0886022X.2016.1193816 27758121

[175] Hishikari K, Hikita H, Nakamura S, Nakagama S, Mizusawa M, Yamamoto T (2017). Urinary liver-type fatty acid-binding protein level as a predictive biomarker of acute kidney injury in patients with acute decompensated heart failure. Cardiorenal Med.

[176] Moledina DG, Isguven S, McArthur E, Thiessen-Philbrook H, Garg AX, Shlipak M (2017). Plasma monocyte chemotactic protein-1 is associated with acute kidney injury and death after cardiac operations. Ann Thorac Surg.

[177] Du J, Cao X, Zou L, Chen Y, Guo J, Chen Z (2013). MicroRNA-21 and risk of severe acute kidney injury and poor outcomes after adult cardiac surgery. PLoS ONE.

[178] Aguado-Fraile E, Ramos E, Conde E, Rodriguez M, Martin-Gomez L, Lietor A (2015). A pilot study identifying a set of micrornas as precise diagnostic biomarkers of acute kidney injury. PLoS ONE.

[179] Ho J, Tangri N, Komenda P, Kaushal A, Sood M, Brar R (2015). Urinary, plasma, and serum biomarkers’ utility for predicting acute kidney injury associated with cardiac surgery in adults: a meta-analysis. Am J Kidney Dis.

[180] Ranganathan P, Mohamed R, Jayakumar C, Ramesh G (2014). Guidance cue netrin-1 and the regulation of inflammation in acute and chronic kidney disease. Mediat Inflamm.

[181] Devarajan P (2010). Neutrophil gelatinase-associated lipocalin: a promising biomarker for human acute kidney injury. Biomark Med.

[182] Vandenberghe W, Loor J, Hoste E (2017). Diagnosis of cardiac surgery-associated acute kidney injury from functional to damage biomarkers. Curr Opin Anesthesiol.

[183] Kim KS, Yang HY, Song H, Kang YR, Kwon J, An J (2017). Identification of a sensitive urinary biomarker, selenium-binding protein 1, for early detection of acute kidney injury. J Toxicol Environ Health A.

